# Non-Contact Monitoring on the Flow Status inside a Pulsating Heat Pipe

**DOI:** 10.3390/s20205955

**Published:** 2020-10-21

**Authors:** Yang Chen, Yongqing He, Xiaoqin Zhu

**Affiliations:** 1 School of Chemical Engineering, Kunming University of Science and Technology, Kunming 650500, China; chenyangzgtc@163.com (Y.C.); xiaoqinzhu@sohu.com (X.Z.); 2 Chongqing Key Laboratory of Micro-Nano System and Intelligent Sensing, Chongqing Technology and Business University, Chongqing 400067, China

**Keywords:** pulsating heat pipe, electromagnetics, magnetic fluids, magnetism theory, sensors

## Abstract

The paper presents a concept of thermal-to-electrical energy conversion by using the oscillatory motion of magnetic fluid slugs which has potential to be applied in the field of sensors. A pulsating heat pipe (PHP) is introduced to produce vapor-magnetic fluid plug–slug flow in a snake-shaped capillary tube. As the magnetic fluid is magnetized by the permanent magnet, the slugs of magnetic fluid passing through the copper coils make the magnetic flux vary and produce the electromotive force. The peak values of induced voltage observed in our tests are from 0.1 mV to 4.4 mV. The effects of the slug velocity, heat input and magnetic particle volume concentration on the electromotive force are discussed. Furthermore, a theoretical model considering the fluid velocity of the working fluid, the inner radius of the PHP and the contact angle between the working fluid and the pipe wall is established. At the same time, the theoretical and experimental results are compared, and the influences of tube inner radius, working fluid velocity and contact angle on the induced electromotive force are analyzed.

## 1. Introduction

The pulsating heat pipe (PHP) is a new type of high-performance heat exchanger, which was first proposed by Akachi [1] and considered as a promising thermal management solution for high heat flux electronic devices and heat recovery systems [2,3,4]. Due to the strong heat transport capacity and self-sustained oscillatory flow inside a PHP, the self-sustained pulsating vapor–liquid two-phase flow can be produced when a sufficiently high-temperature gradient is established between the two ends of the device.

Flow visualization shows that the flow inside a PHP can be random, oscillatory and circulatory [5,6]. Seol et al. [7] and Tong et al. [8] observed that the main flow pattern in an operating PHP is vapor–liquid plug–slug flow. The intermittent vapor plug and liquid slug alternately move upward and downward between the evaporator and the condenser section. Khandekar et al. [9,10] revealed that the heat input is the primary parameter defining the flow pattern, and the pulsating frequency increases with the heat input. The above experimental results were observed through visual experiments of a PHP made of glass.

When it comes to some applications, the shape of the PHP will be more complex, and the structural strength and thermal conductivity of the PHP will be more demanding. Burban et al. [11] designed a kind of PHP radiator for the heat dissipation of electronic equipment in an automobile engine. When the appropriate working fluid is selected, the radiator has considerable heat dissipation performance and can effectively reduce chip temperature. Compared with the traditional PHP when applied in the field of a collector, a flower-type PHP designed by Chen et al. [12] could effectively reduce the footprint of collector.

When the experiment is carried out with a copper tube rather than a PHP which is made of transparent materials such as glass, the movement of the working fluid (liquid slug and vapor plug) in the tube cannot be captured and observed with a camera. Besides, compared with the PHP made of glass, the experimental method with the PHP made of copper is limited. At present, whether the PHP has started can only be judged by the fluctuation of the wall temperature [13]. To solve this problem, an experimental method based on the principle of electromagnetic induction is proposed to monitor the motion of working fluid in a copper PHP.

There are two effects induced by two-phase flow, the magnetic force effect and the magnetic electricity effect, and Kamiyama and Ishimoto [14] proposed a thermal conversion system to enhance the boiling two-phase flow of magnetic fluid under a non-uniform magnetic field. A magnetic repulsion force resulting from the magnetic susceptibility between vapor bubbles and magnetic fluids was used to accelerate the departure of the bubbles.

The electromagnetic induction is conventionally produced by the motion of an electrical conductor in an external magnetic field to cause changes in magnetic flux over time. Since the magnetic fluid is a stable colloidal suspension of single-domain magnetic nanoparticles, which maintains a liquid state with magnetism like liquid oxygen, nitric oxide and platinum [2,4], it is expected that the nonmagnetic phase moving inside the magnetic fluid can induce the electromotive force [3,4,7]. As for the magnetic electricity effect, it means that the millivolt-level voltage could be induced by the magnetic flux disturbances of movements of non-magnetic gas flowing inside magnetic fluids, like the experiment designed by Flament et al. [4], and the value of peak voltage is about 2 mV.

A PHP possesses the abovementioned property since that it is a kind of heat exchange equipment that relies on the phase change of working fluid and transfers heat in the form of latent heat and sensible heat. If the temperature difference between the condenser section and the evaporator section is large enough, continuous vapor–liquid two-phase flow can be generated in the PHP. It can be determined whether the PHP has started according to whether there is an induced electromotive force in the coil. Compared with the method of using a thermocouple to determine whether the PHP is started or not, the time lag of this method is shorter.

Besides, the velocity of the working fluid will affect the value of the induced electromotive force. According to a precise theoretical model, the velocity of working fluid in the tube and the length of working fluid (liquid slug and vapor plug) can be obtained by the induced electromotive force and the time difference between adjacent induced electromotive force peaks. This method will become a new experimental method besides the visual experimental method.

Besides, many pieces of production equipment have strict requirements for the temperature difference of materials. For example, in the field of chemical production, there are often strict requirements for the temperature difference of various reactants involved in the reaction, and the magnetic fluid PHP generator possesses a promising prospect for application in this field. The magnetic fluid PHP is applied to the sensor field without increasing energy externally, which can greatly improve the reliability of the sensor.

Obviously, when the magnetic fluid is used as the working fluid, the magnetic fluid itself will affect the heat transfer performance of the PHP. Maziar et al. [15] used magnetic fluid as the PHP working fluid, and the experiment showed that with the application of a magnetic field, the thermal resistance of the PHP is significantly reduced, and using the ferrofluid in the absence of a magnetic field enhances the thermal performance compared with distilled water. In the horizontal mode, the start-up and operation of the PHP will be negatively affected due to the absence of gravity, but the magnetic force can play the role of gravity, which makes the ferrofluidic PHPs have a favorable thermal performance because of the magnetic force exerted on the ferrofluids. In addition, considering the characteristics of magnetic fluid, the magnetic fluid flow can be controlled by the external magnetic field, which makes it possible to control the heat transfer of the PHP with magnetic fluid as working fluid [16].

In this paper, we demonstrated that the thermally driven magnetic fluid slug flow can lead to the variation of magnetic flux through the induction coils and generate a considerable electromotive force. We also discussed the influence of velocity of vapor plug, heat input and magnetic particle volume concentration on the voltage. An alternating induced voltage with a peak value from 0.1 mV to 4.4 mV was obtained.

The waveform of induced voltage can reflect the flow states inside the pipes, and the influences of two-phase flow patterns and heat input on the waveform, amplitude and frequency of induced voltage are investigated experimentally. Thus, a theoretical model considering the fluid velocity of the working fluid, the inner radius of the PHP and the contact angle between the working fluid and the pipe wall is established, and the influence of the inner radius of the PHP and contact angle between the PHP wall and working fluid on the electromotive force are also discussed.

## 2. Theoretical Considerations/Investigation

### 2.1. Basic Principle

If there is no vapor bubble or plug in the magnetic fluid, the magnetic flux *ϕ* in the coils is:

(1) $$\phi =BS=\mu_{0}\left(M+H\right)\pi d^{2}/4,$$

where *B* is the magnetic induction, *S* is the cross-section area, *μ*_0_ is the vacuum permeability (4*π* × 10^−7^ H/m) and *d* is the diameter of the tube. If there is no other phase in the magnetic fluid under a constant magnetic field, the magnetic field intensity *H* and the magnetization *M* are constant. In this situation, there is no electromotive force due to the magnetic flux on the derivative of the time *dϕ*/*dt*, which is zero.

In Equation (1), the magnetization *M* of magnetic fluid can be calculated by the classical law of Langevin:

(2) $$\frac{M}{\varphi M_{d}}=\coth\zeta -\frac{1}{\zeta },\zeta =\frac{\pi }{6}\frac{\mu_{0}M_{d}Hd^{3}}{k_{B}T},$$

where 𝜑 is the volume fraction of Fe_3_O_4_ nanoparticles, *M_d_* is the saturation moment of the bulk magnetic solid (4.46 × 10^2^ kA·m^−1^), ζ is the Langevin parameter, *k_B_* is the Boltzmann constant (1.381 × 10^−23^ J·K^−1^) and *T* is the absolute temperature.

As the vapor bubbles/plugs pass through the coil, the magnetic flux will no longer remain constant because a portion of space is occupied by non-magnetic vapor. We defined the cross-section fraction *α*, which represents the ratio of the liquid area to the total cross-sectional area. Thus, the expression of the magnetic flux in this case is:

(3) $$\phi =\mu_{0}\left(M+H\right)\alpha \pi d^{2}/4+\mu_{0}(1-\alpha )H\pi d^{2}/4,$$

Since *α* changes with time, its derivative of time could be nonzero. According to Faraday’s law of induction, the expression of induced electromotive force *ε* can be written as:

(4) $$\varepsilon =-n\frac{d\phi }{dt}=-n\frac{d\alpha }{dt}\mu_{0}M\pi d^{2}/4,$$

where *n* is the number of turns of the coils.

From Equation (4), we know that the electromotive force is proportional to the number of turns of the coils, the magnetization of magnetic fluid and the cross-section fraction on the derivative of time. Additionally, the magnetization is the function of temperature under the non-adiabatic condition. Therefore, the electromotive force is mainly determined by the change in the cross-section fraction *α* in our study.

### 2.2. Situations in a PHP

**Figure 1:**
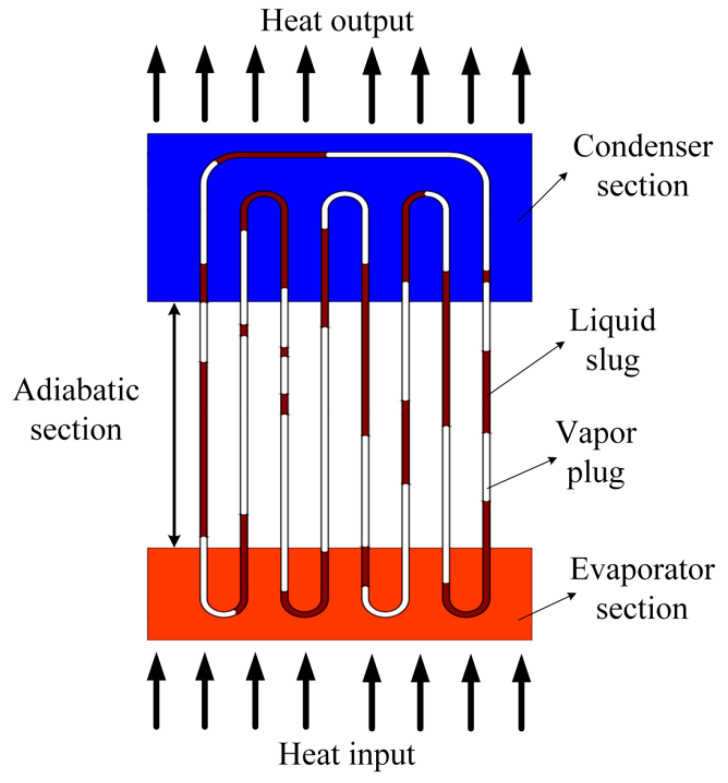
Schematic of the typical structure of a pulsating heat pipe (PHP).

**Figure 2:**
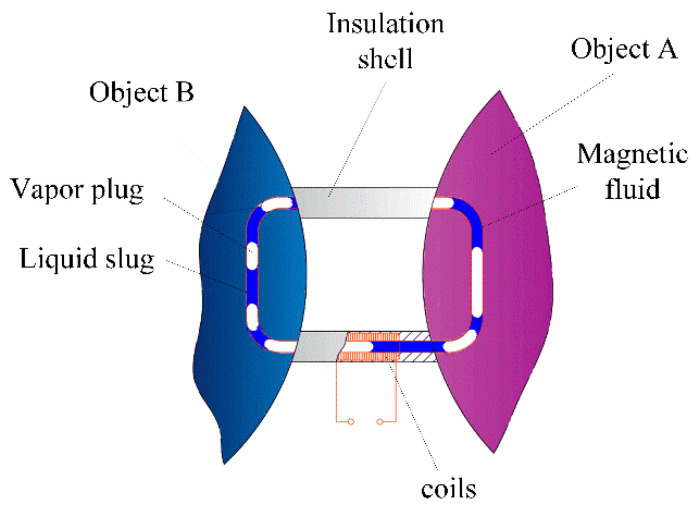
Schematic of a sensor used for monitoring temperature difference.

As shown in Figure 1, a PHP is composed of three parts: evaporator, adiabatic section and condenser. The temperature of the evaporator section continues to rise until the critical heat flux makes the PHP start up. According to this principle, when the PHP is filled with magnetic liquid, it can be used as a sensor to detect the temperature difference between objects, as shown in Figure 2.

**Figure 3:**
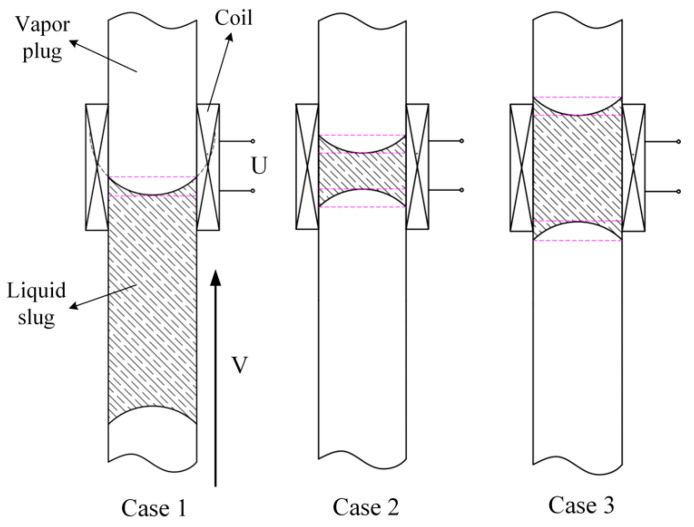
Three situations in which a liquid slug passes through a single coil.

Since the PHP is made of copper, the oxidation of the inner wall of the tube is hydrophilic with the water-based magnetic fluid. Based on this, we assume that the gas–liquid contact surface is an arc. As mentioned above, the induced electromotive force is mainly determined by the change in the cross-section fraction *α*. When the gas–liquid contact surface enters the coil, an induced electromotive force will be generated in the coil. However, due to different lengths of the liquid column, three typical cases for the liquid slug passing through the coil appear in the experiment, as shown in Figure 3.

**Figure 4:**
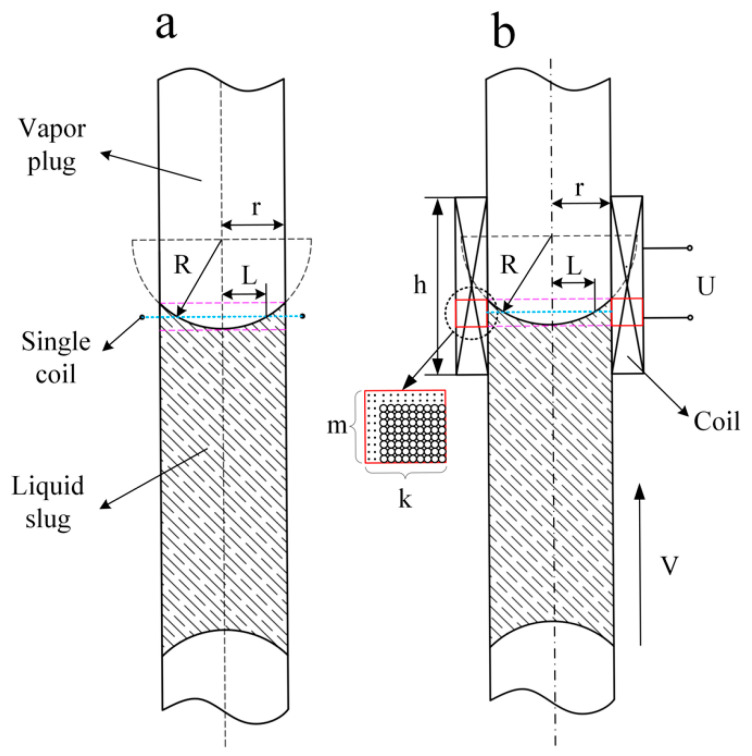
Schematic diagram of theoretical calculation model of induced electromotive force: (**a**) Liquid slug passing through the single coil; (**b**) liquid slug passing through the multi-turn coil.

This paper establishes a theoretical model for case 1, as the liquid slug passes through a single coil, as shown in Figure 4a, and the cross-section fraction *α* can be given by Equation (5) as:

(5) $$\begin{array}{l} \alpha =\frac{S_{1}}{S_{2}}=\frac{\pi \left(r^{2}-L^{2}\right)}{\pi r^{2}}=\frac{{\left(Vt\right)}^{2}+2\sqrt{R^{2}-r^{2}}Vt}{r^{2}},\\ L=\sqrt{R^{2}-{\left(Vt+\sqrt{R^{2}-r^{2}}\right)}^{2}}, \end{array}$$

*S*_1_ is the section area of liquid working fluid in the cross-section, *S*_2_ is the cross-section area of the PHP.

Substituting Equation (5) into Equation (4), the electromotive force can be written as:

(6) $$\begin{array}{l} \varepsilon =-n\frac{d\alpha }{dt}\mu_{0}M\pi d^{2}/4\\ =-\frac{n\left(2V^{2}t+2\sqrt{R^{2}-r^{2}}V\right)\mu_{0}M\pi d^{2}}{4r^{2}}=-f(t), \end{array}$$

where *R*, *r*, *r*_0_ are the radius of the liquid–liquid contact surface, PHP and the wire, respectively, and *V* is the speed at which the liquid slug moves toward the coil. We can see from Equation (6) that the electromotive force ε is a function of *t* (ε = f(*t*), when *t* = 0, *L* = *r*), when the liquid slug moves at a constant speed.

For multiple coils, we assume that each coil is arranged in a square and, as the liquid slug moves, the coil in the red box generates electromotive force, as shown in Figure 4b. In this case, the electromotive force can be calculated by Equation (7).

(7) $$\varepsilon =-k\sum_{i=0}^{m}f(t-it_{0})t_{0}=\frac{2r_{0}}{V},k=\frac{s}{2r_{0}},m=\frac{R-\sqrt{R^{2}-r^{2}}}{2r_{0}}.$$

## 3. Experimental Setup

### 3.1. Structure of PHP

**Figure 5:**
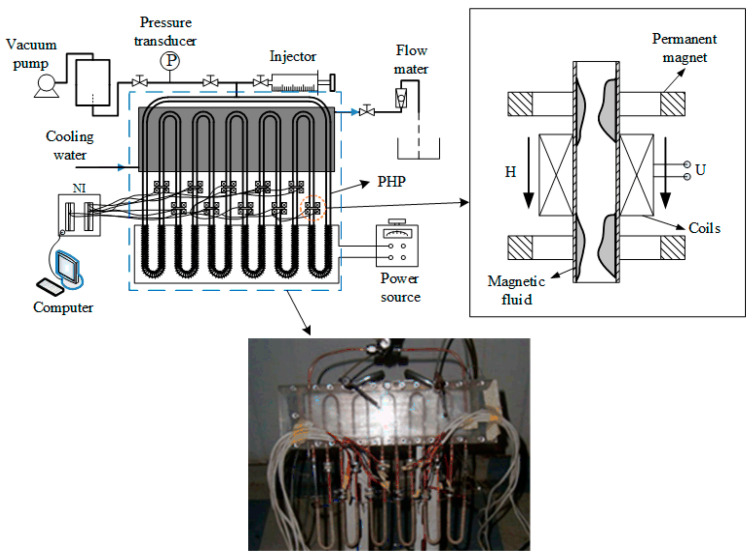
Schematic of the whole experimental system.

The schematic of the experimental set-up is shown in Figure 5. The PHP is made of a brass tube whose outer diameter and inner diameter are 3 mm and 1.5 mm, respectively. The diameter of the tube must be sufficiently small so that capillary action can form vapor plugs. As heat is added to the evaporator region, magnetic fluid is vaporized, causing the vapor volume expansion. Simultaneously, vapor in the condenser region is condensed into liquid causing volume contraction, and the oscillating motion of the liquid slugs and vapor pugs in the snake-shaped tube occurs.

There are 11 elbows in the PHP. The upper, middle and lower parts of the PHP are heating, adiabatic and cooling zones, respectively. The outer wall of the heating section is intertwined with the heating wires. The data collection system collects the parameters, such as the temperature of the cooling water and environment, pressure and current of the heating wire.

Before starting the experiment, the PHP is vacuumed into 400 Pa (absolute pressure). Then the valve is closed before the vacuum pump and a certain amount of magnetic fluid is injected into the PHP. The angle of inclination is maintained at 60 degrees, and the filling ratio is 40% (the heat transfer performance is at a high level with a filling ratio of 30%~40%). The heat input range is from 60 W to 180W with an interval of 30 W.

The inlet temperature and flow rate of cooled water are maintained at 20 degrees and 0.1 kg/min. The PHP is partially filled with magnetic particle volume concentrations of 1.91% and 3.83% water-based magnetic fluids, and the open-circuit voltage of each power generation unit is recorded in the test.

### 3.2. Power Generation Units

There are 10 power generation units composed of two Nd-Fe-B permanent magnet rings and an induction coil, and the structure is shown in the inset of Figure 5. The diameter of the wire is 0.13 mm, and the number of turns is 500. The magnetic field produced by the circular magnets is perpendicular to the PHP and parallel to the induction coils. The two-phase flow of vapor and magnetic fluid cause the change of magnetic flux in the coils and lead to the electromagnetic force.

The values of induced voltage are measured by a National Instruments (NI^®^ SCXI-1000 series) data collection system. The magnetic fluids used in the experiment are made by the chemical coprecipitation preparation method [17], and the surfactant is tetramethylammonium hydroxide 25% aqueous solution. The volume concentrations of Fe_3_O_4_ nanoparticles are 1.91% and 3.83%, respectively.

## 4. Results and Discussion

**Figure 6:**
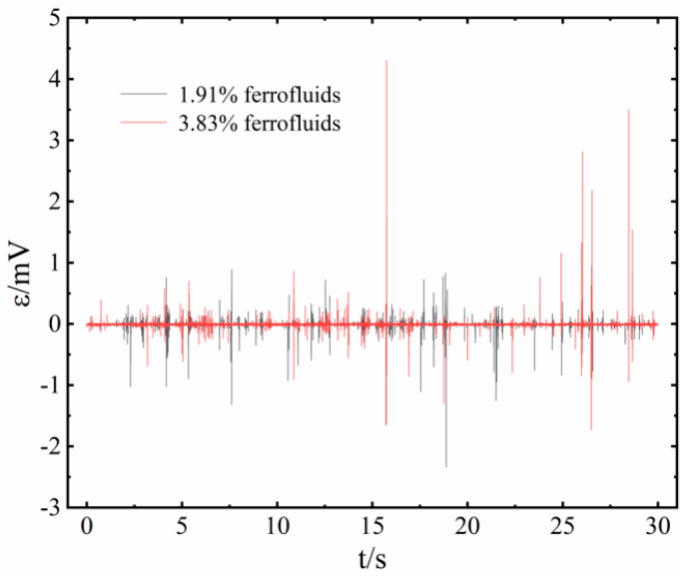
The output voltage of three working fluids in the heat input of 90 W.

Figure 6 shows the output voltage waveforms of power generation unit no. 3 with 90 W power of the heat input, as the PHP was filling with distilled water, with 1.91% and 3.83% magnetic fluids, respectively. The figure shows the data for 30 s, nearly 15,000 points, which are enough to prove the feasibility of this concept. The actual period of voltage fluctuation is less than 20 ms, close to 1500 cycles for 30 s, which leads to the sharp line-shaped figure. The difference in the peak value of the induced voltage is noticeable, and the fluctuation of different peaks exceeds an order of magnitude, which indicates the complexity and randomness of the flow pattern in the tube of the PHP itself. We also checked the total voltage value as connecting multiple units located in the same section of the adiabatic section. No apparent linear growth of voltage was observed, but the average and peak values were higher than the single unit, and the frequency increased a lot.

Obviously, a 3.83% magnetic fluid has a higher peak value because of its higher saturation magnetization.

**Figure 7:**
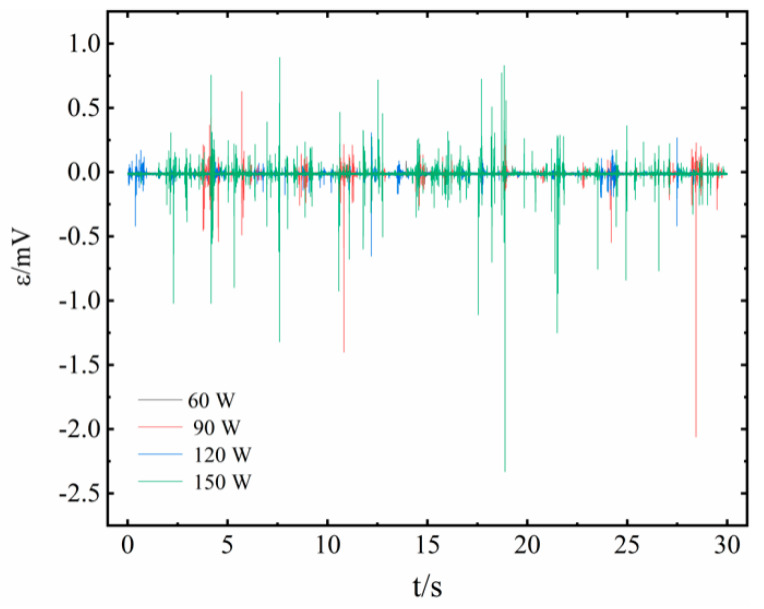
The induced voltage signal with different heat input.

Figure 7 describes the effects of heat input on the induced output voltages of unit no. 3 for the case of 1.91% magnetic fluid. There was almost no induced voltage when the power of heat input was 60 W, which means that there is no pulsating motion inside the PHP as the heat input is too small to lead to the expansions of vapor plugs in the heating sections. Only when it reaches a critical threshold triggering the pulsating phenomenon, does the PHP start to work well, with the value usually between 60 W and 90 W.

The induced voltage begins to fluctuate drastically under 90 W heat input, indicating that the vapor plugs/liquid slugs have passed through the coils at high speed, and the maximum peak value is observed to be 2.1 mV, whereas those of the other peaks are still below 0.5 mV, and the velocity of the vapor/liquid plug is maintained at 1.0 m·s^−1^ or less. It can be understood that there is a relaxation process in the contraction and expansion of the vapor plug/liquid slug in the PHP, and it takes time to trigger the rapid pulsation. Accordingly, the process in the PHP is not smooth and continuous.

The frequency and amplitude of induced voltage increase with increasing heat input, which is because the thermal resistance of the PHP decreases as the heat input increases, and the speed and frequency of the magnetic fluid slug pulsation also increase. The value of peak voltage is most significant when the power of the heat input reaches 150 W, and the maximum voltage peak reaches 2.4 mV. Since high heat input will cause the partial burning phenomenon inside the tube, when the heat input was increased up to 180W, the voltage was not improved.

Voltage waveforms of magnetic liquids with magnetic fluid of 1.91% and 3.83% were similar. However, the magnetic susceptibility of 3.83% magnetic fluid is doubled (the saturation magnetization of 1.91% magnetic fluid is half that of 3.83% magnetic fluid, which is 8.5 kA·m^−1^), and the peak value of the induced voltage is also improved. At 150 W heat input, the maximum peak value is 4.4 mV.

**Figure 8:**
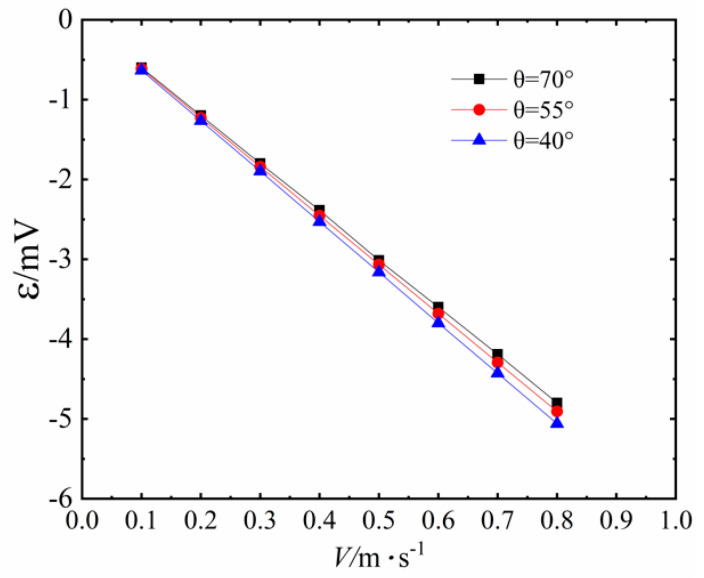
Variation of induced electromotive force with the fluid velocity of working fluid under different contact angles between working fluid and pipe wall.

The visual simulation experiments by Xue and Qu [18] shows that the magnitude is about 0.1–0.7 m/s when PHP works in the state of ‘‘Slug Flow”. The magnitude of fluid velocity is between 0.4–0.9 m/s when the PHP works in the state of ‘‘Annular Flow”. We use the mathematical model to calculate the induced electromotive force generated in the coil as the fluid velocity changes from 0.1 to 0.8 m/s, when the contact angle is 70°, the inner radius of the PHP is 0.75 mm and the volume concentration of Fe_3_O_4_ nanoparticles is 3.83%. The specific values are shown in Table 1 and Figure 8. The induced electromotive force is very close in value compared with the experimental data under the premise of ignoring the contact angle. In addition, there is a standard linear relationship between the velocity of the working fluid and the induced electromotive force.

Figure 8 also shows the variation of induced electromotive force with the fluid velocity of working fluid under different contact angles between working fluid and pipe wall. When the fluid velocity is low, the difference of the induced electromotive force is small in value, and at the same fluid velocity, the induced electromotive force decreases as the contact angle increases. When the fluid velocity is 0.1 m/s, as the contact angle increases from 40° to 70°, the induced electromotive force is only reduced by 5.37%. With the increase in velocity, the difference of induced electromotive force between different contact angles becomes larger and larger in value. However, until the fluid velocity reaches 0.8 m/s, the induced electromotive force is only reduced by 5.14%. This indicates that the contact angle has less and less influence on the induced electromotive force as the fluid velocity increases. We find that the influence of different contact angles on the induced electromotive force is very limited. Thus, even without considering the influence of the contact angle, the theoretical model can reflect the relationship between flow velocity and induced electromotive force well.

**Figure 9:**
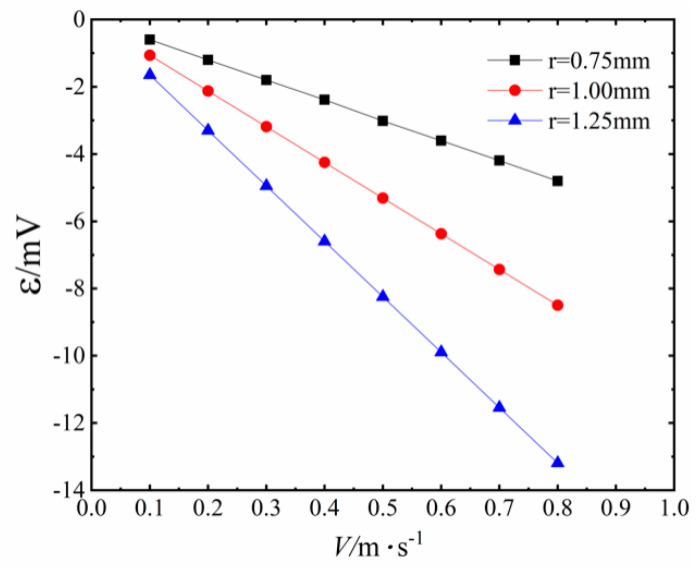
Variation of induced electromotive force with the fluid velocity of working fluid under different inner radii of the PHP.

To study the limit of the induced electromotive force of the PHP, we calculated the influence of different inner radii of the PHP on the induced electromotive force. Figure 9 shows the variation of induced electromotive force with the fluid velocity of working fluid under the different inner radii of the PHP. At different fluid velocities, the induced electromotive force increases proportionately with the increase in the inner radius of the PHP. When the inner radius of the PHP is 1.25 mm and the working fluid velocity is 0.8 m/s, the induced electromotive force is up to 13.191 mV, which indicates that PHP power generation units have great potential in the field of sensors and power generation.

**Figure 10:**
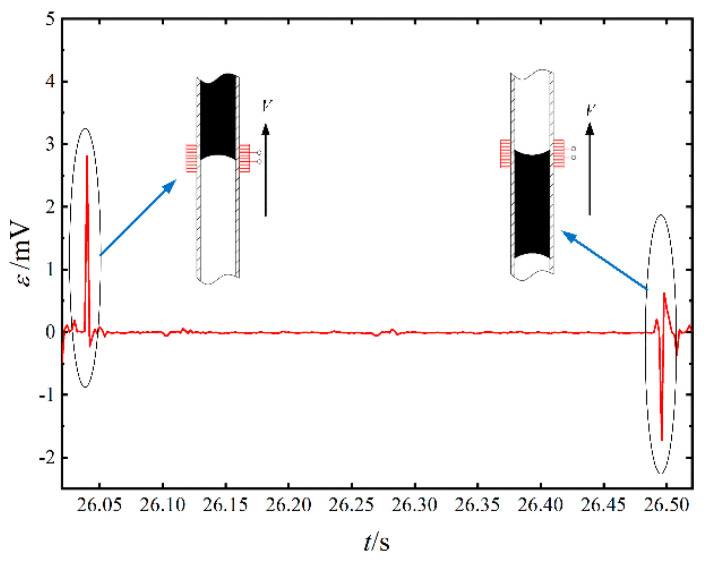
The voltage waveform for 3.83% magnetic fluid at 150 W.

To further reveal the influence of the flow status (flow pattern) inside the tube on the induced electromotive force, there are 0.5 s of data to analyze the waveform. As shown in the right inset of Figure 10, when the phase interface at the top of a liquid slug passed the induced coils, the cross-section fraction increased dramatically and generated the crest of the induced voltage. The velocity of the vapor plug decides the peak value. After the nose leaves the coil area, the tube is full of the liquid so that there is no voltage generated, as shown in the middle inset. The cross-section fraction of liquid is always approximately 1 until the phase interface at the tail of the liquid slug enters the coils. Then, the cross-section fraction of liquid is quickly turned to 0, forming a negative short peak, as shown in the left inset.

## 5. Conclusions

Through further analysis of the theoretical model, the influence of contact angle and pipe diameter on the PHP generator was found, and the electromotive force limit of the generator was studied. As a proof-of-concept demonstration, a PHP which is filled with magnetic fluid with different concentrations has been tested. The device can generate induced voltage with a peak value of 0.1 mV to 4.4 mV. Only when heat input reaches a critical value, could the pulsation phenomenon be triggered. The higher heat input could produce more intense pulsation and stronger induced electromagnetic force. However, dry-out will appear gradually with massive heat input, which may lead to a reduction of induced voltage. The induced voltage waveform can reflect the flow situation in the PHP.

By comparing the experimental data, the theoretical model possesses reliable accuracy, and can accurately calculate the induced electromotive force corresponding to different fluid velocities, pipe diameters and contact angles.

The calculation results indicate that as the inner radius of the PHP increases, the induced electromotive force increases significantly. When the inner radius is 1.25 mm and the fluid velocity is 0.8 m/s, the maximum electromotive force can reach 13.191 mV, which indicates that the magnetic fluid PHP can generate an objective electromotive force under certain conditions.

At a certain fluid velocity, the induced electromotive force decreases as the contact angle increases. However, the size of the contact angle exerts a very limited influence on the induced electromotive force. When the fluid velocity is 0.8 m/s, the induced electromotive force only decreases by 5.14% when the contact angle increases from 40° to 70°.

## Figures and Tables

**Table 1:** Variation of induced electromotive force with fluid velocity.

*V*/(m·s^−1^)	0.1	0.2	0.3	0.4	0.5	0.6	0.7	0.8
*ε*/mV	0.599	1.199	1.800	2.386	3.014	3.600	4.191	4.800
